# Extrachromosomal circular DNA: biogenesis, structure, functions and diseases

**DOI:** 10.1038/s41392-022-01176-8

**Published:** 2022-10-02

**Authors:** Ludi Yang, Ruobing Jia, Tongxin Ge, Shengfang Ge, Ai Zhuang, Peiwei Chai, Xianqun Fan

**Affiliations:** 1 Department of Ophthalmology, Ninth People’s Hospital, Shanghai JiaoTong University School of Medicine, Shanghai, 20025 P. R. China; 2 Shanghai Key Laboratory of Orbital Diseases and Ocular Oncology, Shanghai, 20025 P. R. China

**Keywords:** Cancer genomics, Cancer genomics, Genome

## Abstract

Extrachromosomal circular DNA (eccDNA), ranging in size from tens to millions of base pairs, is independent of conventional chromosomes. Recently, eccDNAs have been considered an unanticipated major source of somatic rearrangements, contributing to genomic remodeling through chimeric circularization and reintegration of circular DNA into the linear genome. In addition, the origin of eccDNA is considered to be associated with essential chromatin-related events, including the formation of super-enhancers and DNA repair machineries. Moreover, our understanding of the properties and functions of eccDNA has continuously and greatly expanded. Emerging investigations demonstrate that eccDNAs serve as multifunctional molecules in various organisms during diversified biological processes, such as epigenetic remodeling, telomere trimming, and the regulation of canonical signaling pathways. Importantly, its special distribution potentiates eccDNA as a measurable biomarker in many diseases, especially cancers. The loss of eccDNA homeostasis facilitates tumor initiation, malignant progression, and heterogeneous evolution in many cancers. An in-depth understanding of eccDNA provides novel insights for precision cancer treatment. In this review, we summarized the discovery history of eccDNA, discussed the biogenesis, characteristics, and functions of eccDNA. Moreover, we emphasized the role of eccDNA during tumor pathogenesis and malignant evolution. Therapeutically, we summarized potential clinical applications that target aberrant eccDNA in multiple diseases.

## Introduction

DNA was discovered by Friedrich Miescher in 1869 and chromosomes consisting of linear double-stranded DNA are known to be the major carrier of genetic material in the eukaryotic nucleus.^1^ Extrachromosomal circular DNA (eccDNA) is a collective name for the circular, double-stranded molecules in the nuclei, which is derived from but independent of chromosomal DNA (chrDNA), with various sizes and sequences.^2,3^ EccDNA can be as small as dozens of base pairs (bp) with only noncoding repeats, while some could contain DNA fragments up to several megabase pairs and even acquire all the genetic elements required for its replication and propagation of the genetic material.^4,5^ The existence of eccDNA is such a common phenomenon that it has been found in all tested eukaryotes, including plants,^6–10^ nematodes,^11^ ciliates,^12^ yeast,^13,14^ drosophila,^15,16^ *Xenopus,*^17,18^ pigeons,^19^ and mammalian species.^20–22^ It is widely distributed in human normal tissues, cancerous tissues, and body fluid.^5,23–25^

Despite being discovered for a long time, the significance of eccDNA remained enigmatic. Moreover, several studies have captured some of the crucial features and functions of eccDNA (especially those with larger structures and functional genes in cancer), which have been validated by advanced high-throughput sequencing technologies. Surprisingly, unlike liner DNA in the chromosome, eccDNA is featured with elevated chromatin accessibility (open-chromatin) and ultralong-range chromatin contact.^2,23,26,27^ Additionally, frequent amplification of tumor-related genes is observed in the eccDNA.^2,28–30^ These features enable eccDNA to drive malignant transformation, promote tumor evolution, and thereby serve as potential biomarkers for tumor diagnosis and prognosis. More importantly, the elimination of extrachromosomally amplified proto-oncogenes has triggered efficient therapeutic efficacy, functioning as a potential strategy in dealing with malignancies.^3,4,31,32^

In this review, eccDNA was introduced from a historical perspective. Subsequently, we discussed the biogenesis and features of eccDNA and summarized the different types of eccDNA, with a focus on their critical roles during multiple physiological and pathological conditions, especially in cancers. We also highlighted up-to-date research methods, tools, and databases in the exploration of eccDNAs. Finally, we provided novel insights into the potential applications of eccDNA in dealing with malignancies.

## History of eccDNA

EccDNA was first reported more than five decades ago. Franklin Stahl conjectured the potential existence of circular DNA in higher organisms, which was later confirmed by Yasuo Hotta and Alix Bassel in 1964. They found circular DNA in mammalian cells, boar sperms, and wheat embryos.^33^ Before long, extrachromosomal DNA elements of diverse sizes and numbers were observed in mitotic human tumor cells. They were referred to as double minutes (DMs) at that time, as they usually appear in pairs.^34,35^ Since then, researchers have focused on updating the understanding of these extrachromosomal particles. From the late 1970s to the 1980s, the existence of eccDNA was confirmed in various types of cancers and cancer cell lines.^36^ The Schimke group was the first to establish the connections between unstable dihydrofolate reductase gene (*DHFR*) amplification and DMs in methotrexate (MTX)-resistant cells. This report provided a glimpse into the biological functions of extrachromosomal DNA (ecDNA), one type of eccDNA that will be discussed further below.^37,38^ Oncogene amplifications, such as *MYC* and *EGFR*, were found in DMs as well.^39–42^ A work on drug resistance of glioma made ecDNA obtain great attention in 2014. Oncogenic *EGFRvIII* was found to primarily reside on extrachromosomes in glioblastoma (GBM). The dynamic elimination and reappearance of oncogene-amplified ecDNA contribute to targeted therapy resistance.^43^

**Fig. 1:**
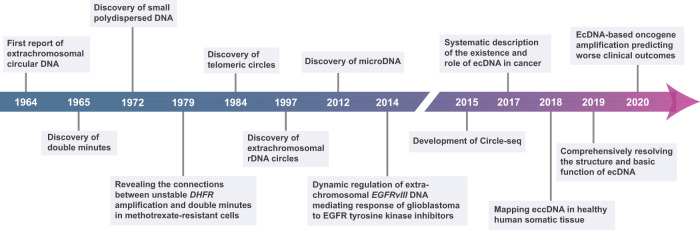
Timeline of the milestone discoveries and research advances of eccDNA. After the first discovery of eccDNA in 1964, multiple categories of eccDNA in various species have been identified. With the development of high-throughput sequencing technologies, the biological properties and functions of eccDNA have been deeply investigated

Technological advancements, particularly next-generation sequencing and bioinformatics, have substantially broadened our knowledge of eccDNA.^44^ Whole-genome sequencing (WGS), along with optimized sequencing data analysis methods, provides opportunities for mapping the landscape of eccDNA in both human cancer and normal tissues. Systematic studies revealed that ecDNA is common and abundant in cancer, although its levels are highly heterogeneous.^45–49^ Additionally, eccDNA widely exists in healthy human tissues and blood.^50^ New highly sensitive methods for detecting and characterizing eccDNA, such as Circle-seq, were subsequently developed and used in investigating the yeast genome.^14,51^ In 2019, researchers rigorously validated the circular structure of ecDNA and evaluated its chromatin state.^2^ Recently, the clinical impacts of ecDNA-based oncogene amplification have demonstrated that patients with tumors containing ecDNA have poorer survival outcomes (Fig. 1).^46^

## Biogenesis of eccDNA

EccDNAs are generated in multiple ways, following a context-dependent manner. Although the precise mechanisms are enigmatic, several models of eccDNA formation have been proposed. The formation processes of eccDNA are generalized into four categories: homologous recombination (HR), nonhomologous end-joining (NHEJ), DNA replication, and the formation of R-loops.^23,26–28,30^ HR and NHEJ are two major repair pathways of DNA double-strand breaks (DSBs).^52^ Studies have shown that HR can excise repetitive DNA sequences during early development and generate eccDNAs such as extrachromosomal rDNA circles (ERCs) and telomeric circles (t-circles/c-circles).^13^ Furthermore, depletion or inhibition of key proteins in NHEJ could trigger a decrease in the amount of eccDNAs, indicating that NHEJ is involved in the formation of eccDNA.^53,54^ In addition, polymerase slippage at the short direct repeat sequence gives rise to DNA loops in the process of DNA replication, and subsequent excision of the loop leads to the formation of eccDNAs.^55,56^ Alternatively, the formation of the R-loop in the process of transcription allows the direct repeats on the unpaired strand to form into a loop which can be excised and ligated into a circle.^55^ Moreover, the widely accepted models for the specific ways in which eccDNA is formed include the breakage-fusion-bridge (BFB) cycle, chromothripsis, episome model, and translocation-deletion-amplification model.^4,27,28,57^

### Breakage-fusion-bridge (BFB) cycle

**Fig. 2:**
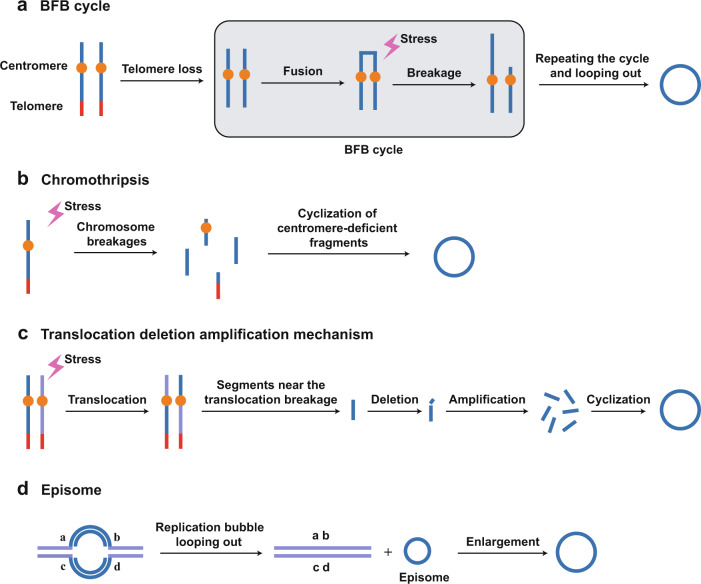
Mechanisms of eccDNA formation. **a** “BFB cycle” model. A dicentric anaphase bridge forms in the absence of telomeres. The BFB cycle involves an anaphase bridge, followed by bridge breakage under stress, releasing eccDNA. **b** “Chromothripsis” model. Single- or double-stranded DNA breaks are formed under exogenous stress, and some of the fragments are ligated and circularized into eccDNA. **c** “Translocation-deletion amplification” model. Exogenous stimuli trigger chromosomal translocation. The DNA segments near the translocation breakpoints are amplified, deleted, and circularized to form ecDNA. **d** “Episome” model. Episomes are produced by DNA slippage and R-loops during the DNA synthesis process and are able to self-replicate and enlarge to form multimerized eccDNA

BFB, first conceptualized by Barbara McClintock in the 1930s, is one model of the genome rearrangement process.^58,59^ The loss of telomeres can cause end-to-end chromosome fusions, forming a dicentric chromosome and developing into an anaphase bridge.^60^ This telomere-free bridge can be extended by replication and randomly broken into fragments under stress, followed by chromothripsis (discussed below) or another BFB cycle. BFB cycles can result in genome instability and the release of eccDNAs (Fig. 2a).^26,61–63^

### Chromothripsis

Another model is the chromothripsis model, where chromosomes are broken into pieces by a catastrophic event.^64^ Most of the DNA fragments can be removed by the DNA repair system, while some of the fragments can be ligated randomly (Fig. 2b).^60,65^ The proposal of the model was prompted by the observation of complex rearrangement in a patient with chronic lymphocytic leukemia. Fragments may be tethered together to drive the formation of ecDNAs carrying oncogenes by accident, which might be tumorigenic.^65–67^ Similar phenomena were reported in oligodendroglioma and esophageal squamous cell carcinoma.^68,69^ Vogt et al. found that the small fragments on the chromosome were associated with contigs in DMs. Fluorescence in situ hybridization (FISH) and WGS revealed various junctions associated with fusions between noncontiguous sequences in the normal reference genome.^68^ Furthermore, studies suggest that DNA damage, which is linked to the chromothripsis model, is involved in the biogenesis of eccDNAs. Mehanna P et al. found that eccDNAs are significantly induced by chemotherapeutic-induced apoptosis in lymphoblastoid cells.^70^ In addition, Sunnerhagen P et al. demonstrated that the carcinogen, 7,1-dimethylbenzoanthracene, the DNA replication inhibitor, hydroxyurea (HU), and the protein synthesis inhibitor, cycloheximide, all promote eccDNA production.^71^

### Translocation-deletion-amplification mechanism

The translocation-deletion-amplification mechanism results from exogenous stimuli and can be removed via the DNA repair system. EccDNAs are derived from retained or cleaved DNA fragments formed during DNA damage repair (Fig. 2c). This model is supported by the coamplification of *MYC* and *ATBF1* in SJNB-12 cells, where a reciprocal translocation occurs between chromosomes 8 and 16, followed by excision and deletion near the translocation breakpoint. The isolated sequences are amplified and circularized to form eccDNAs.^68^ In addition, the translocation-deletion-amplification mechanism is also well suited to account for the coamplification of *HMGIC* and *MDM2* accompanied by t(10;12)(p15;q15) translocation in precancerous pleomorphic adenoma carcinomas.^72^

### Episome model

The episome model is one of the classic models for the biogenesis of eccDNA, where eccDNAs are produced by DNA slippage and R-loops during the DNA synthesis process. These eccDNAs are also named episomes. Episomes are able to self-replicate and can be expanded by incorporating other DNA components, such as transposable elements (TEs) and enhancers/promoters (Fig. 2d). Storlazzi CT et al. showed that *MYC*-containing DMs in leukemia cases are triggered by excision and amplification, which underpins the episome model. Additionally, they also investigated ten cell lines from solid tumors and demonstrated that the *MYC*-containing ecDNAs are derived from excision and amplification as well, which expands the applicability of the episome model to solid tumors.^73^ Furthermore, the formation of *EGFR*-containing ecDNAs results in the generation of cancer-associated circular *EGFR* amplicons, contributing to the oncogenic activation of EGFR.^42^ As the production of ERCs depends on DSB formation at the replication fork barrier (RFB), it is possible that ERCs are released from combined fork breakage at two neighboring replication forks.^74^

## Molecular structures of eccDNA

Since the discovery of eccDNA, emerging studies have systematically analyzed the structure of eccDNA.^75–77^ Advances in next-generation sequencing technologies and computational analysis technologies have revealed several key structural features of eccDNA as follows: First, eccDNAs are circular and independently replicate outside of chromosomes.^2,78,79^ A head-to-tail configuration in the nucleotide sequence was detected through polymerase chain reaction and mapping by restriction enzyme digestion of eccDNA in a human neuroblastoma cell line.^80^ A recent study from late 2019, which combined DNA sequencing and high-resolution imaging, obtained definitive evidence of the circular shape of eccDNA.^2^

Second, eccDNAs vary widely in size, from a few dozen base pairs to hundreds of thousands of base pairs. The sizes and features of eccDNAs vary in different life stages and tissues. Fetal-derived eccDNAs are shorter and hypomethylated compared with maternal eccDNAs.^25,81^ The methylation density of eccDNA is positively correlated with its size. Recent studies have shown that most eccDNAs in normal cells are less than 1000 bp in length. Cancer cells have larger eccDNAs than normal cells (usually greater than 1 kb), and these eccDNAs are long enough to carry the full-length region for the amplification of oncogenes.

**Fig. 3:**
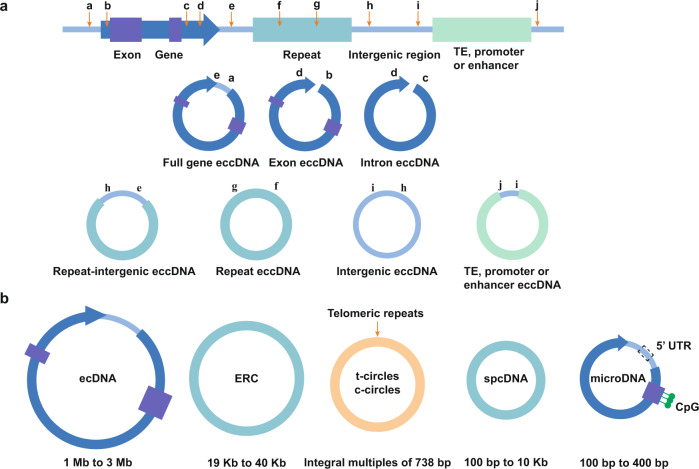
Structures and biological features of eccDNA. **a** Based on genomic origin and genetic content, eccDNAs are categorized into the following types: full-gene eccDNA, exon eccDNA, intron eccDNA, repeat eccDNA, repeat-intergenic eccDNA, intergenic eccDNA, TE, promoter or enhancer eccDNA. **b** Based on size and sequence, eccDNAs are categorized into the following types: small polydispersed DNA (spcDNA), microDNA, telomeric circle (t-circle/c-circle), extrachromosomal rDNA circle (ERC), and extrachromosomal DNA (ecDNA). The main elements and structures of eccDNAs are shown

Third, eccDNAs have different genetic contents, which constitute the structural diversity of eccDNAs.^19,82^ According to their genomic origins and genetic contents, eccDNAs can be categorized into the following eight types: full-gene eccDNA, exon eccDNA, intron eccDNA, repeat eccDNA, repeat-intergenic eccDNA, intergenic eccDNA, TE eccDNA, and promoter/enhancer eccDNA (Fig. 3a). The structural diversity and topological structure of eccDNA contribute to the versatility of its functions by possibly driving the expression of coding RNAs, noncoding RNAs, and other RNAs.^26^

Notably, compared to chrDNAs, eccDNAs mainly contain active histone marks with a more accessible chromatin landscape.^2,5,83^ Immunofluorescence analysis of active and repressive histone marks and H3K4me1/H3K27ac ChIP-seq analyses of GBM39 cells detected active histone marks on ecDNA in GBM39 cells. After normalizing for DNA copy number, transcription was still at a high level, suggesting that ecDNA has a highly accessible chromatin state, which may contribute to the high transcription levels.^2,46^

In addition, eccDNAs appear to possess unusual structural plasticity. Evidence suggests that eccDNA is able to reintegrate into chromosomes and form homogeneously staining regions (HSRs). A large number of factors can cause eccDNA to reintegrate as HSRs, such as DNA damage, doxorubicin treatments, and pharmacological poly ADP-ribose polymerase (PARP) trapping.^67,84^ Studies have identified two major mechanisms by which eccDNA is reintegrated into HSRs. (1) EccDNA segments are reintegrated into chromosomes and amplified through BFB cycles, leading to HSR formation. (2) HSRs can also be formed by reintegration of a multimerized eccDNA originating from the episomes.^85,86^ Importantly, the reintegration of eccDNA into HSRs facilitates enhanced stability and overexpression for these eccDNA-containing oncogenes. For example, HU treatment can eliminate oncogene-containing DMs but cannot decrease the copy number of oncogenes amplified on HSRs.^87^ Furthermore, Richard P Koche et al. found eccDNA fragments reintegration without HSR formation, indicating that eccDNA can also be reintegrated into chromosomes in the absence of HSRs.^88^ Additionally, reintegration of eccDNA may lead to gene misregulation. Drug resistance-related genes can be reintegrated into chromosomes via eccDNA, allowing tumor cells to acquire drug resistance.^38,89–91^ EccDNA inhibits the expression of tumor suppressor *DCLK1* by inserting itself into its gene body, while eccDNA also promotes oncogene *TERT* expression by integrating itself into the vicinity of oncogenes.^88^

## Classifications of eccDNA

Based on size and sequence, eccDNAs can be categorized into the following five types: small polydispersed DNA (spcDNA), microDNA, t-circle/c-circle, ERC, and ecDNA. These are described in detail below (Table 1) (Fig. 3b).

**Table 1:** The unique characteristics and functions of different types of eccDNAs

Classification	Size	Common distribution	Function	Reference
SpcDNA	100 bp to 10 kb	Unstable cells	Contribute to genomic instability	^92–97^
MicroDNA	100 bp to 400 bp	Tumor cells	Serve as regulators in a diverse spectrum of biological processes	^21,70,82,98,99^
Telomeric circles	Integral multiples of 738 bp	ALT cells	Contribute to telomeric maintenance and cell proliferation	^100–106^
ERC	19 kb to 40 kb	Normal cells	Contribute to ribosomal RNA transcription	^56,74,107–109^
EcDNA	1 Mb to 3 Mb	Tumor cells	Contribute to oncogene amplification and genetic heterogeneity	^2,46,47,68,88,113–115^

*EccDNA* extrachromosomal circular DNA, *SpcDNA* small polydispersed DNA, *ERC* extrachromosomal rDNA circles, *EcDNA* extrachromosomal DNA, *ALT* alternative lengthening of telomeres

### Small polydispersed DNA (spcDNA)

SpcDNA is an obsolete concept to commonly characterize small eccDNAs that are between hundreds of bp to a few thousand bp and measure 0.05 to 2.00 μm.^92^ SpcDNA was first observed in the electron microscope examination of the closed DNA from unfractionated HeLa cells in 1967.^93^ From the 1980s to the 1990s, repetitive sequences were widely detected in spcDNAs,^94–96^ and therefore, it is speculated that spcDNA mainly originates from repetitive regions in the genome.^92^ Studies have shown that spcDNA can be found in a variety of eukaryotic cells. SpcDNA is much more abundant in genetically unstable cells and tissues, such as HeLa cells, fibroblasts of Fanconi anemia, and initiating carcinogen-treated cells.^97^ The occurrence of spcDNA has been shown to be linked to genetic instability.

### MicroDNA

MicroDNA, with an average length of 100 to 400 bp, is derived from unique non-repetitive genomic regions with high gene density. It is enriched in the 5′-untranslated regions of genes, exons, and CpG islands.^82^ In terms of distribution, microDNAs are ubiquitous in normal cells of every species, from yeast to humans.^98^ Studies have shown that microDNA levels are dependent on microhomology-mediated end-joining (MMEJ), inhibited by the c-NHEJ pathway, and stimulated by DNA damage.^99^ Further study demonstrated that an increase in microDNA size was observed in human lymphoblastoid cell lines (LCLs) treated with two chemotherapeutic drugs compared with their nontreated counterparts, which suggested a preferential origin of microDNAs from metabolically “active” chromatin sites.^70^ A recent study demonstrated that tumor cells could release specific microDNAs into circulation, indicating that microDNAs may have a role as an attractive biomarker for monitoring cancer progression and therapeutic efficacy.^21^

### Telomeric circle (t-circle/c-circle)

Telomeric circles, as a special type of eccDNA, are duplex (t-circle) or single-stranded (c-circle), consisting only of telomeric repeats. They are integral multiples of 738 bp sequences. T**-**circles occur in a wide range of organisms, including yeasts, plants, and animals. Various DNA damage-associated proteins may regulate the production of t-circles.^100–105^ For example, t-circle reduction by knockdown of the *Ku70/80* heterodimer caused a significant decrease in cell growth in SaOS2 osteosarcoma cells.^106^

### Extrachromosomal rDNA circle (ERC)

ERCs have an average size of 19.3 to 40.4 kb. ERCs can be produced by intramolecular HR of chromosomes and function as templates for ribosomal RNA transcription.^56,107,108^ They are much more abundant in healthy tissue.^74^ Additionally, ERCs can self-replicate due to their autonomously replicating sequences.^109^ ERCs are involved in copy number variations. *Saccharomyces cerevisiae* is able to respond to copy number loss with the clonal amplification of ERCs from chromosomal repeats. ERCs reinsert themselves into the genome in a dosage-dependent manner in response to catastrophic gene loss.^74^

### Extrachromosomal DNA (ecDNA)

EcDNA was first discovered as paired small chromatin bodies in 1964 and was referred to as DMs.^35^ DMs were first found in metaphase neuroblastoma cells and subsequently found in numerous types of cancers.^36,110–112^ With the combined applications of WGS, structural modeling, and computational and cytogenetic analysis, Turner et al. analyzed 17 different cancer types and showed that only 30% of ecDNA in tumor cells presents with DMs-like features. As this group of eccDNAs can either be detected in a double-body form or a single-body form, the definition of these extrachromosomal particles needs to be broadened. Therefore, the term ecDNA refers to those gene-containing extrachromosomal particles of DNA with a size range from 1 to 3 Mb, including both DMs and single-body forms.^2^

EcDNA lacks centromeres and segregates randomly or asymmetrically during cell division. Gene sequences on ecDNA are highly rearranged and amplified. It integrates multiple regions scattered throughout different chromosomes in tumors.^47,113^ In addition, breakpoints were found to be randomly distributed around oncogenes.^46^ Hence, ecDNA is unlikely to have a unified genome template. It may originate from several early genomic events in a random manner and be selectively chosen during tumor evolution.^47^ Furthermore, structural analysis demonstrates that ecDNA evolves constantly via further fusion, rearrangement, and mutation, which increases genomic diversity from another aspect.^68,88,114,115^

## Regulatory mechanisms of functional eccDNA

### EccDNA contributes to gene amplification and signaling pathway regulation

**Fig. 4:**
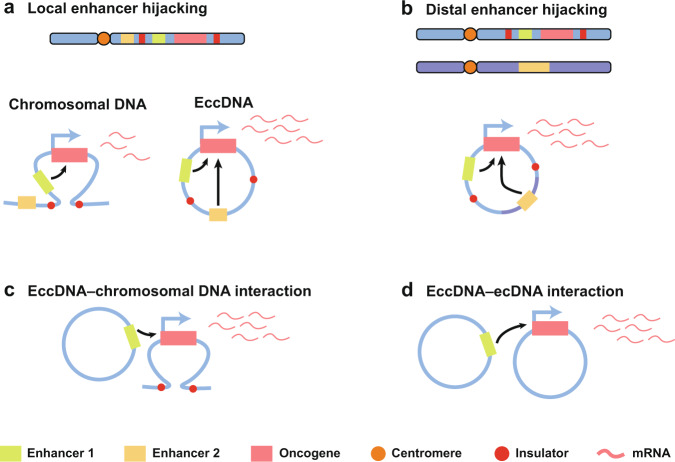
EccDNA enables distal DNA interactions. **a** Oncogene segments and enhancers from adjacent topologically related domains can join together into a circle. **b** EccDNA integrates oncogene segments and distant enhancers to form an ecDNA circle. **c**, **d** EccDNA serves as a mobile enhancer that interacts with both chromosomal DNA (**c**) and other eccDNA (**d**)

Gene amplification is a common molecular alteration in nearly all kinds of cancers, and it provides cancer cells with selective growth advantages.^5,116^ EccDNAs provide an effective method for gene amplification by increasing the copy number directly or acting as trans-acting factors, such as super-enhancers^2,45,117,118^ (Fig. 4). In yeast, eccDNAs are common, and their accumulation and loss affect the copy number of genes carried.^74,119^ This will help yeast respond rapidly to selective pressures and adapt to changing environments.^14,119^

EccDNAs can exert vital biological functions through regulated signaling pathways that influence oxidative stress, bystander effects, and tumor progression.^32^ EccDNA contributes to radiation-induced bystander effects by involving stress signaling. Irradiation affects not only the cells traversed by the radiation track but also non-irradiated neighboring cells, a response described as radiation-induced bystander effects. Several studies have revealed that eccDNAs released from dying irradiated cells can serve as a stress signal that conveys a bystander effect. After the initial irradiation, the irradiated cells executed apoptosis and released oxidized eccDNA fragments, which induced sustained activation of oxidative signaling pathways. Oxidized eccDNA fragments will further interact with recipient bystander cells, causing secondary oxidative stress in bystander cells. In turn, these cells initiate an apoptotic cascade leading to the release of oxidized eccDNA.^120–127^

In addition, eccDNA involves in the regulation of many tumor-related signaling pathways, such as the p53 pathway, MAPK/ERK pathway, Ras, and PI3K/AKT pathway. Zhao X et al. performed functional enrichment analyses through CircleBase, an integrated resource and analysis platform for human eccDNAs. They revealed that cancer cell eccDNAs were mainly enriched in oncogenic pathways such as the Ras and PI3K-Akt signaling pathways.^128^ Studies have shown that eccDNA affects the expression of some important components in signaling pathways. EccDNA can significantly elevate copy numbers of *EGFR*, *MDM2*, *CDK4*, and *BRAF* in tumor cells, which implicates the regulation of the PI3K/AKT pathway, p53 pathway, and MAPK/ERK pathway.^2,129,130^ Moreover, eccDNA can also be regulated by several canonical signaling pathways. Sun et al. found that the stability of DMs in tumor cells is related to ERK1/2 activation. Inhibition of ERK1/2 activation and constitutive phosphorylation of ERK1/2 can significantly reduce the number of DMs and the expression of DM-carrying genes in tumor cells.^131^

### EccDNA participates in restoring telomere length

T**-**circles contribute to the alternative lengthening of telomeres (ALT), which is important in cancer cell proliferation.^132–134^ Tumor cells limit the telomere length by telomere trimming from the chromosome ends, which is mediated by the release of t-circles.^135,136^ Yu et al. detected high levels of t-circles in patients with high-risk neuroblastoma, which are associated with active “telomere trimming”.^137^ Another study revealed that trimming activity in neuroblastoma triggers rapid telomere deletion and increases the number of t**-**circles, promoting extensive proliferation.^138^ Meanwhile, t-circles are involved in the ALT to prevent the shortening of cancer telomeres. Telomerase-negative immortalized human cells maintain their telomeres by ALT. Yeager TR et al. found that ALT cells contain the ALT-associated promyelocytic leukemia (PML) body (APB), which is composed of telomeric DNA and telomere-associated proteins. The presence of APB correlates with the activation of ALT.^136,139–142^

### EccDNA involves in genome plasticity and adaptive evolution

Due to genomic and spatial mobility, eccDNA possesses the capacity for adaptive evolution and genomic plasticity.^57^ Studies have shown that eccDNA is associated with drug resistance by regulating drug resistance-related genes, asymmetrical segregation, or the shift between eccDNA and HSR. *EGFRvIII* oncogenic variant tumor cells are sensitive to EGFR tyrosine kinase inhibitors (TKIs). Tumor cells can reversibly regulate mutant *EGFR* expression, conferring distinct cellular phenotypes to adapt to environmental change. Specifically, GBM cells eliminate mutant *EGFR* from eccDNAs when exposed to TKIs. Moreover, clonal *EGFR* mutation reappears on eccDNAs after drug discontinuation.^43^ In melanoma, the amplifications of *BRAF*^*V600E*^ are highly plastic under MAPK inhibitor treatment through the involvement of *de novo* genomic alterations.^130^

In terms of genomic plasticity, eccDNAs also participate in gene compensation. *HTA1-HTB1* and *HTA2-HTB2* are the two gene pairs that encode histones H2A and H2B. The *HTA2-HTB2* dose compensates at the transcriptional level when *HTA1-HTB1* is deleted.^143^ Libuda DE et al. further revealed that dose compensation of *HTA2-HTB2* occurs by generating an eccDNA carrying *HTA2-HTB2*, the histone H3-H4 locus, a centromere, and the origin of replication when *HTA1-HTB1* is absent.^144^

### EccDNA serves as a molecular sponge

Increasing evidence has proven that eccDNAs can be transcribed into regulatory RNAs that sponge transcription factors. MicroDNAs can be transcribed independently of canonical promoter sequences in vitro and in vivo. As microDNAs are not long enough to carry full protein-coding genes, they usually serve as regulators in a diverse spectrum of biological processes. MicroDNAs express regulatory short RNAs, including microRNAs and novel si-like RNAs, which elicit changes in cell phenotype by regulating gene expression.^98^ Yerlici VT et al. discovered that a large number of eccDNAs were generated during genome rearrangement and that these eccDNAs served as templates for the transcription of rearrangement-specific long noncoding RNAs (lncRNAs).^12^

## Physiological functions of eccDNA

**Fig. 5:**
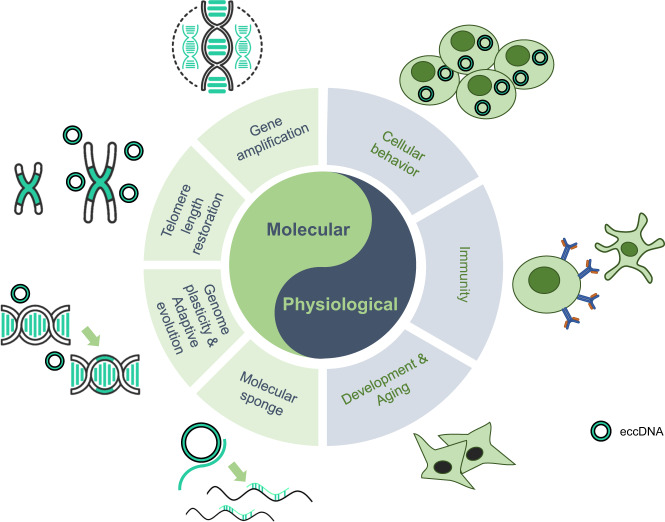
The molecular and physiological functions of eccDNAs. EccDNA regulates diversified molecular and physiological processes. Mechanistically, eccDNA plays an important role in gene amplification, telomere length restoration, genome plasticity, and molecular sponge. EccDNA also plays an important role in multiple physiological events, including cellular behavior, immunity, and aging

Recently, eccDNA has become a hot topic in the scientific field, with increasing publications indicating its involvement in a variety of biological processes. Previous studies have shown that the functions of eccDNAs depend on the gene contents and the structures of the molecular elements. It is necessary to explore the biological functions of eccDNAs to further understand the development and progression of diseases^28,44,56,145^ (Fig. 5).

### EccDNA determines cellular behavior

Studies have shown that eccDNAs affect phenotypes in many ways. It has been widely reported that a high copy number of eccDNA results in oncogene overexpression, which is related to the malignant tumor phenotype. Gemcitabine reduces the amount of DMs in the ovarian cancer cell line UACC-1598, which results in reduced cancer cell growth, colony formation, and invasion.^146^ Furthermore, the proliferative activity of cells was reduced after the elimination of *MYCN*-containing eccDNAs.^147^ EccDNAs also regulate animal phenotypes by reintegrating into chromosomes. Cattle color sidedness (Cs), as a dominantly inherited trait, is determined by homologous yet non-syntenic *Cs* alleles. EccDNAs serve as circular intermediates, contributing to the translocation of *KIT*-containing DNA fragments.^148^ For plants, *EPSPS* gene amplification through eccDNAs confers herbicide resistance to *Amaranthus palmeri.*^149,150^

### EccDNA regulates immunity

EccDNA plays an important role in triggering innate immunity. The superior innate immune-stimulating ability of eccDNA has been detected. EccDNA can regulate the production of proinflammatory cytokines. EccDNA, enriched with CpG-rich genomic DNA fragments, can serve as TLR9 ligands, which increase cell production of IL-6 and TNF-α via activation of the TLR9-MyD88-NF-κB signaling pathway. However, CpG-unenriched eccDNA cannot stimulate the synthesis of IL-6 and TNF-α.^151,152^ As a part of the innate immune response, cells can react to naked DNA in the cytoplasm by activating the cyclic GMP-AMP synthase (cGAS) pathway, leading to stimulation of the immune system. EccDNA may act as an important source of immunostimulatory DNA to activate cGAS-STING innate immune signaling.^153–156^ Furthermore, eccDNAs were able to induce higher levels of cytokines than linear genomic DNA fragments of the same size. When eccDNAs were cleaved into corresponding linear genomic DNA fragments, their strong ability to stimulate an immune response was lost, indicating that the potent immunostimulatory ability of eccDNA depends on its circular structure and cytosolic DNA sensor Sting.^157^ Recently, a study demonstrated that the existence of ecDNA in tumors was negatively correlated with the degree of immune infiltration. Tumor cells may employ ecDNA to decrease immunogenicity and evade immune surveillance.^158^

### EccDNA associates with development and aging

EccDNA is involved in the development of vertebrates. In *Xenopus laevis*, t**-**circles specifically form at the early embryonic stage and are gradually eliminated during normal developmental processes.^17,18,159^ Recent evidence suggests that eccDNA is also associated with aging.^13,44,160^ First, eccDNA accumulates dramatically as cells age in yeast and mammals. Kunisada T et al. analyzed the aging process of rat lymphocytes and human lung fibroblasts and found that the size and copy number of eccDNA increased.^160^ Sinclair DA et al. showed that the accumulation of ERCs is a general phenomenon that occurs in aging yeast cells. These ERCs are able to replicate with an autonomously replicating sequence (ARS), and they are preferentially segregated to mother cells in each cell division. Such asymmetrical segregation results in a marked increase in ERCs in aging mother cells and limits the amount of ERCs in daughter cells. It has been detected that aging yeast mother cells typically exhibit progressive enlargement and fragmentation of the nucleolus due to the accumulation of ERCs.^161^ Furthermore, such accumulation is more obvious after *Sgs1* mutations, but the lack of *Fob1* and *Bud6* will reduce the accumulation of ERCs and extend life.^107,162–164^

## Tools and methods for studying eccDNA

### High-throughput technologies and validation methods

EccDNA could be reconstructed by analyzing WGS data. The detection of extremely amplified and rearranged regions featuring discordant paired-end reads and split reads in the tumor genome hints at the existence of circular DNA, which can be inferred and resolved by bioinformatic analysis tools, such as AmpliconArchitect and AmpliconReconstructor.^2,45,47,113,165^ However, bioinformatics algorithms based on WGS tend to leave out large numbers of low-frequency eccDNA. Circle-seq, a purification and detection method, was developed to screen for a novel or low abundant eccDNA in the genomic range. The research in *Saccharomyces cerevisiae* provides a universal tool for investigating the relationship between copy number variation and eccDNA in other models.^14^ The results of these two methods is highly consistent in large eccDNA. However, small eccDNAs presented with different results according to Circle-seq and WGS. Therefore, it is necessary to integrate results from both WGS and Circle-seq, benefiting a comprehensive and accurate characterization of eccDNA.^46,88^ CIDER-Seq is another novel circular DNA detection method based on randomly primed circular DNA amplification, followed by long-read single-molecule sequencing. Long-read sequencing could overcome the shortcomings of the short-read sequencing methods we aforementioned, which fail to accurately resolve the complicated architecture of ecDNA.^4,166,167^ Nevertheless, this method cannot guarantee the accuracy of detecting eccDNA larger than 8 to 10 kb.^51^ In addition, ATAC-seq data could be reanalyzed by Circle_finder to identify eccDNA.^49^ Other high-throughput methods, especially epigenetic techniques, are quite useful for exploring the topological structure and function of eccDNA. ChIP-seq, PLAC-seq, ATAC-seq, MNase-seq, 4C-seq, and Hi-C have been used to reveal the chromatin accessibility and nucleosome compaction state of eccDNA.^2,166^ Moreover, combined analysis of single nucleotide polymorphisms in the RNA-seq and WGS data could distinguish whether the transcripts are derived from genes on eccDNA or from linear chromosomes.^2^

After prediction, inverse PCR and Sanger sequencing could be adopted to verify the circularity of putative eccDNA. Outward PCR will amplify the desired products if a circular structure does exist. The junction points could be confirmed by aligning the sequencing data to the reference genome.^2^

### Imaging and visualization methods

Microscopic imaging has made a huge contribution to the discovery of eccDNA historically. The presence of mammalian DNA in a circular configuration was first identified by electron microscopy.^33^ Contemporaneously, when examining the karyotype of surgically removed tumor tissues, researchers observed large molecular weight eccDNA, minute double chromatin bodies stained with DNA dye in mitotic metaphase cells under light microscopy.^35^ Recent advances in imaging focus on depicting eccDNA at high resolution by structured illumination microscopy and atomic force microscopy.^63,168^ In addition, Yi E et al. utilized a CRISPR dCas9-based DNA labeling system to visualize the spatiotemporal dynamics of eccDNA in live cells. Single-guide RNA targeting eccDNA-specific breakpoint junctions can introduce fluorescent tags to eccDNA and track the uneven segregation behaviors of eccDNA during mitosis.^169^

**Fig. 6:**
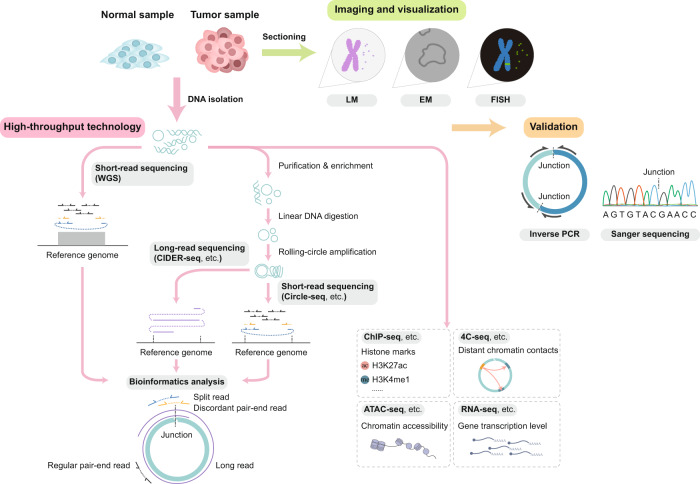
Tools and methods in the exploration of eccDNA. High-throughput technologies have been applied to detect the existence and structure of eccDNA. EccDNAs can be reconstructed by analyzing data from short-read sequencing, including whole-genome sequencing and Circle-seq, along with long-read sequencing by a bioinformatics algorithm. Other epigenetic techniques, such as ChIP-seq, ATAC-seq, and 4C-seq, assist in the exploration of the topological structure. Notably, inverse PCR and Sanger sequencing have been applied in the validation of eccDNA. Light microscopy (LM), electron microscopy (EM), and fluorescence microscopy are involved in the visualization of eccDNA and relevant genetic amplifications

The links between copy number variation and eccDNA are often neglected. Compared with genome sequencing, which has high sequence resolution, FISH can visually show the intracellular position of target genes. It is contrary to common sense that many highly amplified oncogenes actually reside in eccDNA instead of linear chromosomes.^43,169^ Furthermore, advanced tools, namely, ECdetect and ecSeg, for analyzing microscopic images have been developed recently (Fig. 6).^45,78^

### EccDNA resources

With the rapid development of high-throughput sequencing and bioinformatics analysis technologies, it has been shown that eccDNAs are widespread in human disease. This illustrates the need to establish eccDNA-related databases. Zhao et al. constructed a public database, CircleBase for the annotation and functional analysis of eccDNA in various human cells. CircleBase, equipped with highly interactive eccDNA visualization capabilities, can identify functional eccDNAs by combining sequencing datasets, computational predictions, and manual annotations and provide comprehensive eccDNA annotation.^128^ Peng et al. constructed a database of eccDNA profiles in human cancers named “eccDNAdb”. This database provides not only basic information and annotations of eccDNA but also the prognostic value of eccDNA genes.^170^

## EccDNA in human cancers

**Fig. 7:**
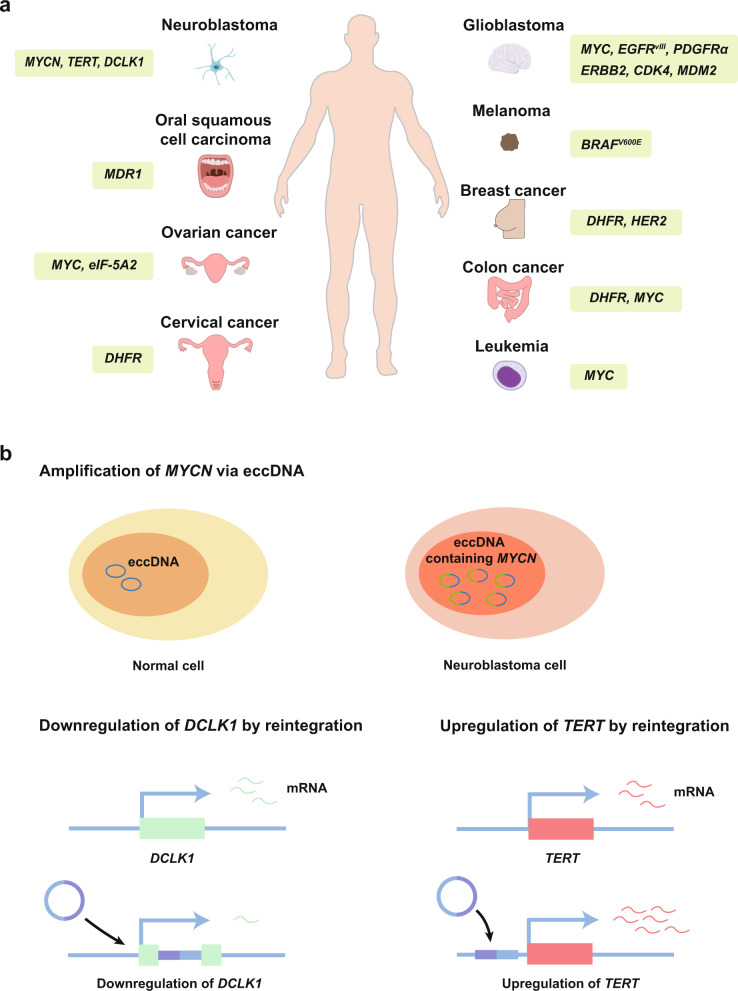
EccDNA in human cancers. **a** EccDNA and its containing genes in human cancers. In neuroblastoma, *MYCN* encoded by eccDNA is associated with tumor progression. EccDNA reintegrates into chromosomes in neuroblastoma and affects chromosomal gene expression of *TERT* and *DCLK1*. In glioblastoma, *MYC*, *EGFR*, *PDGFRα*, *ERBB2*, *CDK4*, and *MDM2* are amplified via eccDNA. The mutation of *EGFR* amplified by eccDNA is dynamically regulated to evade therapy. In colon cancer, *DHFR*-carrying eccDNA contributes to MTX resistance. *MYC* is amplified via eccDNA. In breast cancer, *HER2* is amplified via eccDNA, and *PIP* on eccDNA is associated with genetic instability. In cervical cancer, *DHFR*-carrying eccDNA contributes to MTX resistance. In ovarian cancer, eccDNA-containing *MYC* and *eIF-5A2* stimulates tumorigenesis. In leukemia, *MYC* encoded on eccDNA contributes to tumorigenesis. In oral squamous cell carcinoma, loss of *MDR1*-carrying eccDNA promotes drug sensitivity. In melanoma, focal amplifications of *BRAF*^*V600E*^ contribute to tumorigenesis and drug resistance. **b** EccDNA facilitates the oncogenesis of neuroblastoma in different ways. EccDNA serves as a template for directing the transcription of *MYCN*. EccDNA inhibits the expression of *DCKL1* by inserting it into its gene body. EccDNA promotes *TERT* expression by integrating itself into the vicinity of oncogenes

EccDNA, especially ecDNA, is widespread in nearly half of human cancers, following a context-depedent manner across diversified cancer categories.^45^ The unique structure and molecular characteristics enable a highly spatial and temporal plasticity of eccDNA functions, which determines the pathogenesis of cancer initiation, progression, and evolution.^3,171–173^ (Tables 2, 3 and Fig. 7a).

**Table 2:** Summary of the roles of eccDNA in development

Cancer type	Genes on eccDNA	Functions	Reference
Pancancer	*EGFR, MYC*, *CDK4, MDM2*	intratumoral heterogeneity, copy number alteration	^2,281^
Neuroblastoma	*MYCN*	copy number alteration, distal DNA interaction, reintegration into chromosomes	^199^
Glioblastoma	*MYC, EGFR, PDGFRα, ERBB2, CDK4, MDM2*	copy number alteration, distal DNA interaction	^2,43,259^
Colon cancer	*MYC*	copy number alteration	^249^
Breast Cancer Ovarian cancer	*HER2, PIP* *eIF-5A2, MYC*	copy number alteration, genetic instability copy number alteration	^232,315^ ^146,250^
Leukemia	*MYC*	copy number alteration, reintegration into chromosomes	^147,249^
Melanoma	*BRAF*	copy number alteration, reintegration into chromosomes	^130^

**Table 3:** Summary of eccDNA-related drug resistance

Cancer type	Drug-resistance genes	Drugs or treatment	Functions	Reference
Glioblastoma	*EGFR*	Erlotinib Irradiation	The mutations of *EGFR* amplified by eccDNA are dynamically regulated to evade therapy	^43^
Colon cancer	*DHFR*	Methotrexate	*DHFR*-carrying eccDNA contributes to MTX resistance	^53,266,267^
Cervical cancer	*DHFR*	Methotrexate	*DHFR*-carrying eccDNA contributes to MTX resistance	^316^
Oral squamous cell carcinoma	*MDR1*	Hydroxyurea	Loss of *MDR1*-carrying eccDNA promotes drug sensitivity	^273^
Melanoma	*BRAF*^*V600E*^	Vemurafenib, Selumetinib	Focal amplifications contribute to drug resistance	^130^

*MTX* methotrexate

### EccDNA in cancer initiation

Oncogene amplification, as one of the most common molecular alterations in cancer, plays an important role in tumorigenesis by providing cancer cells with selective growth advantages.^174–179^ EccDNA promotes oncogene amplification and induces cancer initiation by serving as a template for directing the transcription of oncogenes, altering the landscape of regulatory elements, enhancing chromatin accessibility, and inducing genomic remodeling.

#### EccDNA serves as a template for oncogene transcription

Containing more than one full gene and regulatory regions, eccDNA can encode genes, especially bona fide oncogenes. MYC family proteins (MYC, MYCN, and MYCL) are major drivers of human tumorigenesis.^180–183^ MYC proteins regulate the expression of multiple genes and affect multiple biological processes, including cell proliferation, growth, senescence, metabolism, differentiation, and apoptosis.^184–186^ Genome alterations in MYC family genes, particularly gene amplifications, are recognized as early events in a wide variety of cancers, leading to dysregulations of cell functions that result in embryonal or cancer stem-like qualities, such as increased self-renewal, apoptotic resistance, and metabolic flexibility.^187–192^ In neuroblastomas, *MYCN* amplification has been detected in over 20% of neuroblastomas patients.^193–198^ Prior research generally confirms that eccDNA drives copy number amplification of *MYCN* in neuroblastoma. In the 1960s, eccDNA was first discovered in metaphase spreads of neuroblastoma cells. Numerous eccDNAs in the form of DMs were detected, with no evidence of other chromosomal breaks. Subsequently, in the 1980s, NE et al. further observed the amplified DNA sequences from human neuroblastoma cell lines to explore the role of gene amplification in maintaining the phenotype of neuroblastomas. They detected a new oncogene called *MYCN*, whose amplification sequence was located on DMs in the human neuroblastoma cell line IMR-32.^39^ More recently, a genome-wide landscape of extrachromosomal DNA circularization in neuroblastoma confirmed that the *MYCN* gene is amplified on eccDNA in neuroblastoma^199^ (Fig. 7). Moreover, it has been detected that eccDNA contains amplified *MYC* in leukemia.^200–202^ L Abbate A et al. performed a range of high-resolution genomic methods and revealed a large cohort of acute myeloid leukemia (AML) cases harboring *MYC* amplification in the form of DM, HSR, and ring chromosomes.^203,204^ Besides, several studies have also detected *MYC*-containing eccDNA in GBM, colon cancer, and ovarian cancer.^2,147,205^

EGFR is a receptor tyrosine kinase (RTK) that is involved in the regulation of the cell cycle, apoptosis, angiogenesis, and cellular proliferation.^206–209^ *EGFR* amplification has been reported to play an important role in cancer initiation.^210–214^ In GBM, oncogene amplification is the most frequent gain-of-function alteration, which enables tumor cells to circumvent the checks and balances that are in place during homeostasis, thereby driving tumorigenesis.^215,216^ Among them, amplification of *EGFR* is the most common molecular hallmark of GBM, which is detected in approximately 40–50% of primary GBM patients.^217–220^ Wu et al. demonstrated that eccDNAs in the GBM cell lines encode *EGFR* as well as other oncogenes, such as *MYC*, *CDK4*, and *MDM2*, which account for the top 1% of genes expressed in the cancer genome. They further showed that oncogenes amplified by eccDNAs have higher copy numbers than the same genes amplified by linear chromosomes.^2^

Human epidermal growth factor receptor 2 (HER2) plays an important role in cell growth and differentiation.^221^ It affects the activation of the MAPK pathway and PI3K pathway, which are associated with tumorigenicity.^222–225^ Approximately 25% of breast cancers overexpress HER2.^226–231^ Studies have shown that eccDNA is involved in *HER2* amplification in breast cancer. Vicario R et al. detected that *HER2* amplification in DMs or in HSR occurs in 30 and 60% of HER2-positive breast cancers, respectively, indicating that different mechanisms of *HER2* gene amplification exist in breast cancer.^232^

The eukaryotic initiation factor eIF-5A2 has been reported to be an oncogenic protein in multiple human cancers.^233–236^ Accumulated evidence suggests that eIF-5A2 initiates tumor formation, enhances cancer cell growth, and increases cancer cell metastasis.^146,237,238^ Guan XY et al. showed that *eIF-5A2* is amplified in the ovarian cancer cell line UACC-1598 in the form of DMs.^146^ Conclusively, amplification of oncogenes on eccDNA provides a novel theoretical basis for explaining tumorigenicity.

#### EccDNA alters the regulatory element landscapes

In addition to increased copy numbers, transcriptional activity may be the result of alterations in regulatory element landscapes.^2^ The amplicon structure and the chimeric circularization elements can promote oncogene expression through regulation in the noncoding genome.^239^

First, eccDNA may act as a powerful enhancer hijacking element in cancer. In neuroblastoma, Helmsauer K et al. analyzed the *MYCN* amplicon structure and chromatin landscape of ecDNA by using short-read sequencing, Nanopore sequencing, ChIP-seq, ATAC-seq, and Hi-C. They showed two enhancer hijacking models of eccDNA to explain the regulatory requirements for *MYCN* overexpression. In the first type, a proximal enhancer is coamplified, triggered by the noradrenergic core regulatory circuit (CRC). In the second type, *MYCN* amplicons are characterized by the presence of distal chromosomal fragments harboring CRC-driven enhancers.^166^ Likewise, the local enhancer hijacking is also involved in *EGFR* amplification in GBM.^239^ Morton et al. revealed *EGFR* amplicon patterns in eccDNA from primary GBM sequencing. They demonstrated the coamplification of *EGFR* and its upstream enhancer on ecDNA, where the new enhancer–oncogene contacts contribute to cancer cell growth.^240^ Considering that no breakpoints were detected between *EGFR* and its two upstream enhancers, it is suggested that the incorporation of endogenous enhancers is not only advantageous for GBM cell survival, but also required for oncogene selection. It can be concluded that the chimeric circularization can be generated by a selective pressure to amplify proto-oncogenes together with suitable regulatory elements. Moreover, the proto-oncogenes share the same regulatory neighborhood by residing side-by-side on the same DNA. The enhancer hijacking models may extend to other cancers and contribute to identifying relevant loci in the various complex aberrations that drive cancers.

Second, eccDNA serves as a mobile enhancer that interacts with chrDNA or other eccDNA in cancer. Zhu et al. identified various trans-interaction sites using ChIA-Drop chromatin interaction assays and ChIA-PET, which indicates that ecDNA can interact with genome-wide DNA as a mobile enhancer.^83^

In addition, ecDNAs are able to trans-activate the oncogene expression by forming hubs. A recent study showed that the amplification of *MYC* in colon cancer is triggered by ecDNA hubs. These ecDNA hubs are bound by the bromodomain and extraterminal domain protein BRD4 and enable intermolecular enhancer and gene interactions to promote *MYC* overexpression. Inhibition of BRD4 has been shown to have the ability to disaggregate ecDNA hubs and reduce ecDNA-derived *MYC* expression.^205^ The discovery of ecDNA hubs in colon cancer has significant implications for understanding how ecDNA undergoes selection and how ecDNA regulates transcription. Furthermore, it is proposed that ecDNA hubs can effectively recruit a large number of RNA polymerases and transcription factor complexes to form molecular condensates as a separate phase in the nuclei.^83,169^ The concept of “ecDNA-associated phase separation” provides novel insights into oncogenesis.

Furthermore, the function of eccDNA noncoding sequences in oncogene amplification has been characterized. Jin et al. used bioinformatics analysis and revealed that the 682 kb DMs in ovarian cancer harbors five matrix attachment regions (MARs). PCR analysis showed that these MARs can bind to the nuclear matrix in vivo, indicating that they are functional. Measured by luciferase assay, increased oncogene expression was detected following the transfection of MARs constructs, indicating that noncoding regions on DMs regulate gene expression and are involved in oncogene activation.^241^ Ultimately, they concluded that high oncogene expression cannot be achieved by oncogene amplification alone, but with coamplification by MAR elements.

#### EccDNA enhances chromatin accessibility

EccDNA has a highly accessible chromatin state that enables distal DNA interactions and promotes oncogene amplification, which contributes to cancer initiation. Wu et al. demonstrated the presence of active histone marks on GBM39 ecDNA and the absence of repressive histone marks. The results from ATAC-seq and MNase-seq revealed that the chromatin landscape of ecDNA is more accessible than chrDNA due to its less compacted nucleosomal organization.^2^ Studies of eccDNA have uncovered an insightful layer of regulatory complexity in tumorigenicity, which may provide access for the targeted disruption of oncogene amplification.

#### EccDNA involves in genomic remodeling

EccDNA is a major source of somatic rearrangements in various types of cancer, resulting in oncogenic remodeling through reintegration into the linear genome.^88^ Reintegration of eccDNA could cause gene dysregulation, resulting in the defective balance of proto-oncogenes and tumor suppressor genes, which further contributes to tumorigenesis.^242^ In leukemia, Von Hoff DD et al. used gel electrophoresis techniques to locate the amplified *MYC* oncogene sequences in leukemia cells. With increased passages in culture, the amplified *MYC* shifted from DMs to a chromosomal site and was accompanied by a shortened cell doubling time, suggesting that eccDNA actively regulates expression levels by genome rearrangements.^243^ A study in neuroblastoma further showed that the expression of oncogenes such as *TERT* was markedly increased because of the integration of eccDNA into the vicinity of oncogenes, while the expression of the tumor suppressor *DCLK1* was repressed due to the integration of circle fragments into the gene body^88^ (Fig. 7b).

### EccDNA in cancer progression

The accumulation of eccDNAs in cells affects the malignant phenotype of tumors. Notably, patients with more oncogene amplification on eccDNA had significantly worse 5-year survival outcomes, indicating that the abundance of oncogene-containing eccDNA was associated with tumor aggressiveness. Simulation models showed that tumors carrying a circular amplicon have higher cellular proliferation scores and lower immune infiltration scores.^46,244^ Subsequently, a series of studies were carried out to explore the molecular characteristics of eccDNA in tumors. Computational analysis of WGS data performed by Kim H. et al. showed that circular amplicons of ecDNA are more prevalent in aggressive cancers such as GBM.^46^

Notably, studies have demonstrated that elimination of tumor eccDNA reduces oncogene amplification, thereby reverting the tumor malignant phenotype. In neuroblastoma, Ambros IM et al. revealed that extrachromosomally amplified *MYCN* copies can be eliminated from the nucleus in flat cells (F-cells). The reduction of amplified sequences in F-cells results in a decreased proliferative activity and upregulated expression of the major histocompatibility complex class I (MHC I).^31^ In vitro treatment with drugs such as HU induce the micronucleus formation and thus expulsion of amplified genes, triggering efficient anti-cancer efficacy in multiple maligancies.^31,153,245,246^ Recently, neuroblastoma cell lines with DMs exhibited enlarged and flattened morphology and increased granularity, and expressed senescence-associated-β-galactosidase (SA-β-GAL) when exposed to low-dose HU, suggesting that low-dose HU can serve as an effective senescence activator for neuroblastoma cells with DMs.^247,248^

Additionally, DMs in colon cancer can be removed from the nucleus by budding of the nuclear membrane during S-phase. Cell synchronization and bromodeoxyuridine-pulse labeling indicate *de novo* bud and micronucleus formation in S-phase, which are regulated by the p53 pathway. However, it has been further demonstrated that HU treatment eliminated oncogene-containing DMs but did not decrease the copy number of oncogenes amplified on HSRs, which indicates that oncogene amplification is more stable on HSRs, rather than on ecDNAs.^87^ Another study showed that HU is able to eliminate DMs with amplified copies of *MYC* in colon cancer, leading to a reduction in tumorigenicity.^249^ Overall, drugs that promote S-phase budding may be valuable in the treatment of colon cancer. However, the efficacy is limited by the dynamic shifting between DMs and HSR.

In ovarian cancer, Guan et al. studied the correlation between the reduction of *eIF-5A2* copy number and cell growth rate and showed that the cell growth rate was inhibited when the *eIF-5A2* copy number of DMs was reduced.^250^ Yu et al. found that gemcitabine is able to decrease the number of DMs in the ovarian cancer cell line UACC-1598. Cells treated with gemcitabine showed reduced cell growth, colony formation, and invasion, indicating that gemcitabine affects the biology of ovarian cancer cells by decreasing the number of DMs.^251^

Previous studies in leukemia suggested that an independent active process may naturally exist in HL-60 cells to eliminate the extrachromosomal amplification of *MYC* and that this process can be enhanced by drugs including a low dose of HU or dimethyl sulfoxide.^252^ A low dose of HU increases the percentage of spontaneously differentiated cells where the amplification of *MYC* is decreased by entrapment within micronuclei.^147,249^ Understanding the molecular mechanisms of the natural elimination process has important implications for drug intervention in leukemia.

### EccDNA in cancer evolution

#### EccDNA drives tumor heterogeneity

Tumor bulk is a collection of cell populations with genetic, phenotypic, and behavioral heterogeneity.^253,254^ Available evidence suggests that clonal evolution is likely mediated by eccDNA, especially ecDNA. EcDNA is thought to be acentric and unevenly separate into daughter cells at cell division.^169,255–257^ Live-cell imaging was applied during mitosis and showed disjointed ecDNA inheritance patterns.^169^ Single-cell analyses of a patient-based xenograft model showed that the ratio of EGFRvIII^High^/EGFRvIII^Low^ cells in the subcutaneous GBM formed by FACS-sorted EGFRvIII^High^ or EGFRvIII^Low^ seeds was the same. However, *EGFRvIII* heterogeneity caused by ecDNAs was completely different under continuous drug selection. This result suggested a distinct pattern of ecDNAs distribution to daughter cells, which does not follow Mendel’s law of inheritance and thereby create heterogeneous cell populations. Subsequently, specific subpopulations were screened out by an ever-changing external microenvironment to maximize the chances of survival.^43^ A simulation model also predicts that the oncogene copy numbers on ecDNA could rapidly reach to and maintain a highly variable state in this way.^45^ Increased intratumoral genetic heterogeneity will provide tumors with extra survival advantages to cope with external pressure.^43,89,258^

#### EccDNA involves in drug resistance

Studies have shown that changes in the cellular composition mediated by eccDNA facilitate drug resistance and thereby dictate a patient response. In GBM, Nathanson DA. et al. found that resistance to TKIs in preclinical models and GBM patients is related to a decreasing ratio of EGFRvIII^High^/EGFRvIII^Low^ tumor cells. Such regulation is achieved by the elimination and reemergence of *EGFRvIII* from eccDNA.^43^ It provided theoretical foundations for pulsed intermittent treatment with higher EGFR TKI doses in GBM patients to achieve better therapeutic outcomes, as extrachromosomal *EGFRvIII* DNA levels rapidly rise during the treatment interval. Furthermore, Nikolaev et al. created a concept called amplification-linked extrachromosomal mutations (ALEMs), which refers to extrachromosomal mutations that originate extrachromosomally and could be eliminated from tumor cells. ALEMs are most frequently seen in GBM and low-grade gliomas and occur not only in *EGFR* but also in *PDGFRα* and *ERBB2*, which highlights the diversity of mechanisms by which eccDNA is involved in drug resistance to targeted therapies in GBM.^259^ In addition to chemotherapy, eccDNA has also been associated with radiotherapy resistance in GBM. Zhou et al. investigated the function of *EGFR*-encoding DMs in GBM by comparing two syngeneic primary cultures derived from a GBM with and without cells carrying *EGFR*-encoding DMs. Compared to cells without DMs, those with DMs were relatively radio-resistant, with an elevated level of glycolytic respiration. In response to radiation, DMs-containing cells could switch their respiration from glycolic metabolism to oxidative phosphorylation and shift the molecular profile to that of DMs-free cells. After exposure to an irradiated environment, cells with DMs can alter their extracellular microenvironment not only to stimulate the invasiveness of surrounding cells but also to build a pro-angiogenic tumor microenvironment.^260^ It is suggested that DMs-containing GBM cells may lead to tumor recurrence due to the high invasiveness and radio-resistance.

Studies have shown that eccDNA plays a role in colon cancer chemotherapy resistance. MTX, an antifolate chemotherapeutic agent that inhibits DHFR, is widely used in colon cancer treatment.^261–265^ *DHFR* is able to be amplified in the form of DMs or HSR when cells are treated with MTX. Interestingly, in the absence of MTX, the resistant cell lines had reduced copies of the *DHFR* gene in eccDNAs, which suggested that the dynamic regulation of eccDNAs determines the response toward chemotherapy.^53,266,267^ Furthermore, Meng et al. found that the inhibition of DNA-PKcs (a key NHEJ protein) inhibits the formation of *DHFR-*containing eccDNAs and therefore increased MTX sensitivity in colon cancer cells. They revealed that the depletion of eccDNAs serves as a promising strategy in MTX-resistant cancers.^53,232^

Drug resistance is the leading cause of death in cervical cancer patients and has been a major challenge in cervical cancer treatment.^268–271^ Ruiz-Herrera A et al. detected that deficiency in the HR protein RAD54 resulted in a marked increase in DM-containing subpopulations in cervical cancer cell-derived MTX-resistant subclones. They further showed that DMs were the predominant amplified structures observed in MTX-resistant HeLa parental cells and that amplification of *DHFR* on DMs may contribute to MTX resistance.^272^

Another study showed that HU accelerates the specific loss of extrachromosomally amplified drug-resistance genes from vinblastine- and MTX-resistant oral squamous cell carcinoma cells, suggesting that the elimination of eccDNA-containing amplified drug-resistance genes contributes to improving drug sensitivity.^273^

In melanoma, the *BRAF*^*V600E*^ mutation is considered a major oncogenic driver and can be detected in approximately half of the advanced melanomas. Song et al. revealed that *BRAF*^*V600E*^ was amplified by either DMs or intrachromosomal homologous regions in melanoma. They demonstrated that focal amplification of cells harboring *BRAF*^*V600E*^ shows a mode switch between DMs and HSR in response to changes in drug concentration. Focal amplifications can combine with kinase domain duplications and alternative splicing to enhance drug resistance. In addition, melanoma cells with *BRAF-*containing DMs harbor increased resistance towards dual BRAF and MEK inhibition.^130^

Furthermore, cisplatin resistance of hypopharyngeal squamous cell carcinoma is found to be related to eccDNA. *RAB3B* is amplified on eccDNA, which promotes chemoresistance of hypopharyngeal squamous cell carcinoma cells by inducing autophagy.^274^ Recognizing the association of focal eccDNA amplification patterns with drug resistance is important for understanding cancer evolution and drug resistance, providing therapeutic approaches to overcome plasticity.

## Potential applications of eccDNA

### Serving as promising biomarkers

The discovery of nuclear budding and micronucleation provides possible pathways for eccDNA to escape from cells. Due to the extraordinary structural stability of eccDNAs compared to linear DNA, they may be an ideal source of potential biomarkers.^25,28,275,276^ Several studies have demonstrated the potential application of extrachromosomal DNA elements in body fluids as candidate biomarkers for the diagnosis and monitoring of various diseases (Table 4).^81,277,278^

**Table 4:** Summary of potential clinical applications of extrachromosomal circular DNA as a biomarker

eccDNA	Diseases	Detail information	Reference
SpcDNA	colon carcinoma, cervical cancer, ovarian adenocarcinoma, breast adenocarcinoma, hepatocellular carcinoma, malignant melanoma, osteogenic sarcoma, leiomyosarcoma, tuberous sclerosis, angiofibroma	spcDNA can serve as a marker of genomic instability	^20,92,282,287^
MicroDNA	ovarian cancer, lung cancer, chronic kidney disease, fetal growth restriction	microDNA is highly consistent with tumor burden in cancer patients and is enriched in chronic kidney disease.	^21,285,286^
Telomeric circles	ALT^+^ osteosarcoma, B chronic lymphocytic leukemia, malignant glioma neuroblastoma	t-circles/c-circles are highly specific for the ALT-positive tumors	^137,284,317^
ERC	/	/	/
EcDNA	adrenal carcinoma, neuroblastoma, thyroid cancer, cervical cancer, lung cancer, fetal growth restriction	ecDNA can be exploited as reliable biomarkers of tumor progression and is related to tumor recurrence	^280,281,286,316,318^

*EccDNA* extrachromosomal circular DNA, *SpcDNA* small polydispersed DNA, *ERC* extrachromosomal rDNA circles, *EcDNA* extrachromosomal DNA, *ALT* alternative lengthening of telomeres

**Fig. 8:**
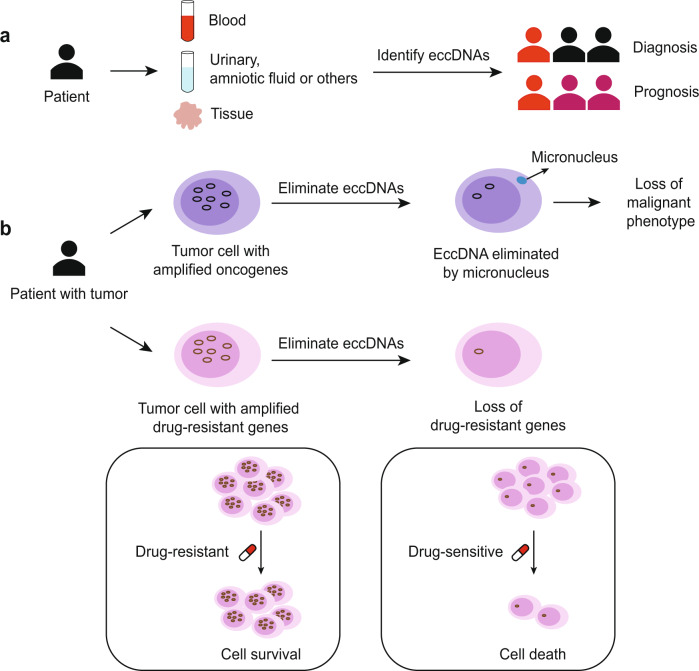
Clinical applications of eccDNA. The clinical value of eccDNAs in cancer. **a** EccDNAs can be found in patient samples, such as blood, urine, amniotic fluid, or tissue. The identification of eccDNAs would be helpful in cancer diagnosis and prognosis. **b** Elimination of eccDNA results in a loss of malignant phenotype. It can also lead to a loss of drug-resistance genes and thereby improve therapeutic effects

EccDNA may serve as a potential biomarker in cancer diagnosis and is valuable in monitoring cancer progression (Fig. 8a). Studies show that cancer patients with ecDNAs seem to have significantly lower survival rates than those without ecDNAs.^279^ The study by Fan et al. found that the frequency of DMs in malignant tumors was much higher than that in benign tumors or noncancerous tissues, suggesting that these DMs can serve as reliable biomarkers for tumor progression.^280^ Furthermore, the abundance of DMs in peripheral blood lymphocytes has been shown to be an independent risk factor in lung cancer patients.^281^

Apart from ecDNA, other types of eccDNA can also act as biomarkers for precision cancer therapy. Cohen et al. demonstrated the potential role of spcDNA as a marker of genomic instability. They showed high levels of spcDNA in genetically unstable cells and tissues.^92^ Moreover, Schmidt et al. showed that Alu sequences and LINE-1 sequences occur frequently in tumor tissues and are dependent on the number of spcDNAs.^282^ Kumar et al. detected tumor-derived human microDNA in a mouse xenograft model of human ovarian cancer. These microDNAs are longer in lung cancer tissue than in normal lung tissue. Longer cell-free microDNA is enriched in preoperative samples compared to postoperative samples.^21^ Unlike other classes of eccDNA species, t-circles/c-circles are highly specific for ALT mechanisms and can be used as promising biomarkers for the diagnosis and management of ALT-positive tumors.^283^ Fogli et al. analyzed 63 malignant gliomas and identified that c-circles were significantly associated with low Ki-67 immunostaining.^284^

EccDNA also serves as an ideal biomarker in other diseases. A study showed that urinary cell-free eccDNAs (ucf-eccDNAs) act as significant biomarkers in advanced chronic kidney disease (CKD). Lv et al. analyzed ucf-eccDNAs from 21 patients with advanced CKD by the Circle-Seq method and demonstrated that ucf-eccDNAs have a distinct disease-specific profile in CKD patients.^285^ Yang et al. performed Circle-Seq to identify eccDNAs in the fetal growth restriction (FGR) group and the normal group. They found that the total amount of eccDNA in the FGR group was significantly higher than that in the normal group and showed a double peak length, peaking at ~146 bp and ~340 bp, respectively. It provided a new vision for the screening of new biomarkers for FGR.^286^ Neidlinger C et al. detected significantly more spcDNAs in the angiofibroma-derived cell cultures of patients with tuberous sclerosis (TS) than in the skin of these patients or in the skin of 11 healthy donors.^287^ Motejlek K et al. further found that the spcDNA from angiofibroma cultures was much longer than that from skin fibroblast cultures. Furthermore, the total amount of spcDNA increased with age in angiofibroma-derived cultures but not in skin fibroblast cultures.^288^

As biomarkers, there are several differences between eccDNA and linear circulating cfDNA, and eccDNA is more advantageous in some respects (Table 5).^289,290^ First, eccDNAs are resistant to exonuclease digestion due to their closed circular structure. Therefore, they are more stable than their linear counterparts.^25,275^ Second, some eccDNAs identified in the circulation, such as microDNA, are much longer than linear DNA, facilitating detection and dynamic monitoring.^21^ Third, eccDNAs have lineage specificity in human ovarian/prostate cancer cell lines and cell type specificity in human fibroblasts and granulocytes.^11,24^ Furthermore, the rolling-circle amplification of eccDNAs can prevent site-specific amplification with specific primers typically used to detect linear cfDNA, which enables the detection of genome-wide circular cfDNA without pre-existing bias in its origin locus.^21^

**Table 5:** The unique characteristics of linear cfDNAs and eccDNAs as biomarkers

Classification	Structure	Resistant to exonuclease digestion	Stability	Length	Amplification	Bias of the origin site
linear cfDNA	Linear	Weak	Unstable	Short	Amplified with specific primers	Exist
eccDNA	Circular	Strong	Stable	Long (in some types of eccDNA, such as microDNA)	Amplified by rolling-circle amplification with random primers	Avoid

*EccDNA* extrachromosomal circular DNA, *CfDNA* cell-free DNA

### Serving as potential therapeutic targets

Carrying complete genes, segregating randomly, and harboring genomic plasticity make eccDNA a potential target for disease treatment^291,292^ (Fig. 8b). DNA replication inhibitors (HU, gemcitabine, and radiation) eliminate eccDNA by integrating eccDNA into cytoplasmic micronuclei, resulting in the silencing of multiple oncogenes.^273,293^ The effectiveness of these DNA replication inhibitors against tumor cells has been verified in various cancers, such as neuroblastoma, colon cancer, ovarian cancer, leukemia, and squamous cell carcinoma.^47,113,153,200^ However, most of these studies have been conducted at the cellular and animal levels. A clinical trial has investigated whether a noncytotoxic dose of HU decreases the number of metaphase spreads containing DMs in tumor cells in patients with ovarian carcinomas.^294^ More clinical studies are needed to further investigate the effectiveness of DNA replication inhibitors in cancer patients.

EccDNAs have been determined to play key roles in the resistance of chemo- or radio-therapy; therefore, the elimination of eccDNA may benefit a better outcome together with conventional therapy.^32,86,295–297^ A large amount of laboratory evidence has shown that tumor cells can reversibly regulate the expression levels of key proteins by altering the amount of eccDNA, conferring different cell phenotypes for drug resistance. In addition, drug resistance may also be induced by creating double-strand DNA breaks, contributing to the conversion of eccDNA to HSR.^67^ Conclusively, DNA repair inhibitors that eliminate eccDNA and inhibit double-strand DNA breaks, combined with conventional therapies such as radiation, chemotherapy, and targeted drugs, have the potential to prevent drug resistance in cancer patients.

## Limitations of eccDNA

Real-time monitoring of eccDNA in circulating blood has provided a scientific basis and new insights for further investigation of the use of these DNA elements as biomarkers for precision cancer therapy. Although the clinical application of the detection of circular DNA in liquid biopsies to identify tumorigenesis and progression is attractive, several challenges remain.

First, there are significant differences in the abundance of eccDNA in different tissues, and the amount of eccDNA in some diseases is not large enough to detect. The inability to provide biopsies of some tissues limits the spectrum of clinical applications.^30^

Second, improvements in eccDNA enrichment and data analysis are needed. Currently, reliable methods to easily quantify the abundance of circular DNA elements are lacking. As the most commonly used method to detect eccDNAs, Circle-Seq amplifies eccDNAs by rolling circles with random primers, which may result in a loss of a significant amount of information related to the abundance of specific eccDNAs.^298,299^ In addition, it lacks a gold standard for eccDNA analysis. Standard analysis algorithms can improve the accuracy and efficiency of eccDNA sequence extraction. A standard analysis and control of bias are needed to make the results more reproducible. The possibility of circulating eccDNA being taken up by cells and the difference in distribution between live and dead cells should be considered in the bias analysis.^21^

Efficient and specific removal of eccDNA is expected to be a new method for cancer treatment in the future. However, most methods for eliminating eccDNAs lack specificity. To date, no technique has been used to remove targetable eccDNA. CRISPR/Cas9 is a common gene-editing tool that uses RNA-guided nucleases to cleave foreign genetic elements.^300–307^ CRISPR/Cas9 may serve as a potential method to target unique eccDNA breakpoint sequences, although it still lacks experimental evidence.^300,301,308,309^ The effects of CRISPR/Cas9 on the elimination of eccDNA need to be further explored. In addition, although HU has shown its ability to eliminate eccDNAs and their contained oncogenes, HU treatment cannot reduce the amplification of oncogenes on the HSR.^310^

## Conclusion and prospects

Chromosomes consisting of linear DNA and histone proteins are the fundamental stores of genetic information in most individuals and species. The discovery of eccDNA has shed new light on spatial information in current genome maps. With rapid advances in the field of eccDNA, many publications have demonstrated that eccDNA functions as an important regulator in intracellular homeostasis and its abnormalities serve as a pathological trigger for diseases. Herein, we summarized all types of eccDNA and demonstrated their functions in multiple biological processes. Furthermore, this review summarized the biogenesis, the biological and pathological functions, databases, related methodology, and the limitation of eccDNA from a historical perspective. Collectively, we introduced the recent progress of eccDNA publications, benefiting an in-depth understanding of this topic for readers. However, some areas still remain to be addressed in this field.

First, what are the specific mechanisms driving the formation and maintenance of eccDNAs? What triggers its reintegration, and where is it reintegrated? As it is known that the stability of DNA is related to the double-helical structure, what is the specific topology of eccDNA? Do topoisomerase participate in the formation and maintenance of eccDNAs? Is there a unified theoretical framework to explain the biogenesis of eccDNAs?

Second, many studies have proposed that eccDNA plays an important role in tumorigenesis. Elimination of eccDNAs reduces oncogene amplification, thereby reverting the tumor phenotypes.^12,110,311^ Studies have shown that noncytotoxic strategies that eliminate eccDNA could contribute to personalizing treatment decisions in cancer patients. It is necessary to clarify the dynamic mechanism for regulating eccDNA quantity. The origin of eccDNAs remains controversial. These non-ecDNA eccDNAs appear to be selected, replicated, and propagated in the cells and are functional regulators in the maintenance of intercellular homeostasis. Does it actively adjust based on the environment, as in Lamarck’s theory, or is it passively selected by the environment, as in Darwin’s theory?^312^

Third, do eccDNAs interact with other biological processes? Several studies have reported the relationships between eccDNAs and epigenetic factors, such as the accessible chromatin landscape and enhancer hijacking. Other epigenetic modification mechanisms of eccDNA, such as DNA and histone modifications, still require further study. Subdivision of the intracellular space coordinates various biological processes in space and time. Phase separation induces the formation of membrane-less compartments to separate intracellular materials.^313,314^ Determination of whether eccDNAs are affected by phase separation requires further exploration and discussion.

Recent studies of eccDNA have forced a reconsideration of the spatial information contained in the genome atlas. EccDNAs have been shown to play specific roles in disease initiation and progression by overriding hereditary constraints and traditional segregation laws. We look forward to new discoveries that will lead to the development of a new fundamental understanding of eccDNA and to advances in clinical diagnosis and treatment.
